# Macrophage M2 polarization promotes pulpal inflammation resolution during orthodontic tooth movement

**DOI:** 10.1111/jcmm.18350

**Published:** 2024-05-03

**Authors:** Jichang Li, Huiying Ren, Zijie Zhang, Jin Zhang, Fulan Wei

**Affiliations:** ^1^ Department of Orthodontics, School and Hospital of Stomatology, Cheeloo College of Medicine Shandong University & Shandong Key Laboratory of Oral Tissue Regeneration & Shandong Engineering Laboratory for Dental Materials and Oral Tissue Regeneration & Shandong Provincial Clinical Research Center for Oral Diseases Jinan Shandong China; ^2^ Department of Endodontics, School and Hospital of Stomatology, Cheeloo College of Medicine Shandong University & Shandong Key Laboratory of Oral Tissue Regeneration & Shandong Engineering Laboratory for Dental Materials and Oral Tissue Regeneration & Shandong Provincial Clinical Research Center for Oral Diseases Jinan Shandong China

**Keywords:** dental pulp, hypoxia, inflammation, macrophage polarization, mechanical force, orthodontic tooth movement

## Abstract

Mechanical force induces hypoxia in the pulpal area by compressing the apical blood vessels of the pulp, triggering pulpal inflammation during orthodontic tooth movement. However, this inflammation tends to be restorable. Macrophages are recognized as pivotal immunoreactive cells in the dental pulp. Whether they are involved in the resolution of pulpal inflammation in orthodontic teeth remains unclear. In this study, we investigated macrophage polarization and its effects during orthodontic tooth movement. It was demonstrated that macrophages within the dental pulp polarized to M2 type and actively participated in the process of pulpal inflammation resolution. Inflammatory reactions were generated and vascularization occurred in the pulp during orthodontic tooth movement. Macrophages in orthodontic pulp show a tendency to polarize towards M2 type as a result of pulpal hypoxia. Furthermore, by blocking M2 polarization, we found that macrophage M2 polarization inhibits dental pulp‐secreting inflammatory factors and enhances VEGF production. In conclusion, our findings suggest that macrophages promote pulpal inflammation resolution by enhancing M2 polarization and maintaining dental health during orthodontic tooth movement.

## 1 INTRODUCTION

Orthodontics is an effective treatment for malocclusion in conditions of maintaining dental health. Orthodontic tooth movement is a complex biological process, which promotes tooth movement by applying mechanical force to periodontal tissues.^1^ Mechanical force simultaneously leads to a series of restorable inflammatory changes within the dental pulp, including increased vascular density, oxidative stress, macrophage accumulation and odontoblast layer alterations.^2^, ^3^ This pulpal inflammation under mechanical force is gradually reduced over time.^4^, ^5^ It allows the health of the orthodontic tooth to be maintained in the end.^6^, ^7^ In order to ensure the health of orthodontic teeth, the potential mechanism of inflammation resolution needs to be further explored.

Existing studies have found that mechanical force can exert pressure on the periapical blood vessels, reducing blood flow and oxygen supply to the pulp.^8^, ^9^ These impacts lead to pulpal hypoxia.^10^, ^11^ Our previous study also confirmed the expression of HIF‐1α and VEGF in dental pulp is enhanced by mechanical force.^12^ These highlighted the critical role of hypoxia signalling in the pulp inflammation during orthodontic tooth movement. Meanwhile, the concept that hypoxia can induce inflammation through the activation of the immune system is widely accepted.^13^ In the dental pulp immune system, macrophages are recognized as pivotal immunoreactive cells.^14^ In particular, they can polarize into different statuses, including classically activated macrophages (M1) and alternatively activated macrophages (M2),^15^ which exert pro‐inflammatory and anti‐inflammatory effects.^16^, ^17^ This allows macrophages to play a dual role in homeostasis maintenance by triggering immune responses, promoting inflammation or promoting healing, clearing apoptotic cells and regulating stem cells.^18^, ^19^

Whether macrophage polarization is influenced by hypoxia and involved in the resolution of pulpal inflammation in orthodontic teeth remains unclear. To address this knowledge gap, we proposed the hypothesis that macrophages are involved in the regression of pulpal inflammation during orthodontic tooth movement through transforming polarization state. We validated this hypothesis by ex vivo and in vivo experiments. The results show that pulpal macrophages gradually shift towards an anti‐inflammatory state (M2) in orthodontic teeth. These macrophages induced by hypoxia can promote the expression of anti‐inflammatory factors and VEGF by dental pulp cells. This transformation of macrophage polarization contributes to the resolution of pulpal inflammation.

## 2 MATERIALS AND METHODS

### 2.1 Rats orthodontic tooth movement model

We utilized 20 seven‐week‐old male Wistar rats (Charles River) weighing around 220 g. Rats were maintained under a 12‐h day‐night cycle with unrestricted access to food and water. We ligatured the left maxillary first molar of each rat to their upper incisor using a nickel‐titanium closed‐coil spring secured by a 0.25 mm stainless steel thread (TOMY, Japan). Approximately 20 g of force was applied. The rats were divided into five random groups based on the duration of dental device maintenance (1, 3, 7, 14 and 28 days), with each group comprising four independent samples for statistical analysis. The right side was taken as the control. After the injection of 3% pentobarbital sodium, the left tooth as a model of tooth movement and their alveolar bone were collected and fixed in 10% formalin for 24 h. The bone tissues were decalcified in 14% ethylenediaminetetraacetic acid (EDTA) for 3 months. The paraffin‐embedded tissues were cut into 5 μm thick slices and then dehydrated with an alcohol gradient.

### 2.2 Cell culture

RAW264.7 cells simulating M0 macrophages in dental pulp were obtained from Procell Life Science and cultured in DMEM high glucose medium (10% FBS, 1% penicillin/streptomycin) at 37°C with 5% CO_2_. Logarithmically growing cells were chosen for experiments under normal (37°C, 5% CO_2_, 21% O_2_) and hypoxic conditions (2% O_2_) using a C‐chamber incubator (ProOx P110 O_2_ Controllers, BioSperix). Mouse Dental Pulp Cells (MDPCs) representing pulp tissue cells were isolated from maxillary bilateral first molars were extracted from 6‐ to 8‐week‐old mice. MDPCs were cultured with normal conditions (37°C, 5% CO_2_, 21% O_2_) and hypoxic conditions (2% O_2_) for 24 h using a C‐chamber incubator (ProOx P110 O_2_ Controllers, BioSperix). To assess the anti‐inflammatory effects of M2 polarization of macrophages under hypoxia, we used trametinib to inhibit their M2 polarization to study its effect on MDPCs. The M2 inhibition group was treated with 10 nM trametinib under 37°C, 5% CO_2_, 2% O_2_ for 24 h, and which were maintained using the same C‐chamber incubator (ProOx P110 O_2_ Controllers, BioSperix).

### 2.3 RNA Extraction and Quantitative Reverse‐Transcription Polymerase Chain Reaction (qRT‐PCR)

Total RNA was extracted using TRIzol (Takara, Japan) following the manufacturer's instructions. The cDNA reverse‐transcription process utilized the PrimeScript RT Reagent Kit with gDNA Eraser (Takara). Following cDNA synthesis, qRT‐PCR was performed in triplicate on the LightCycler 480 system (Roche Diagnostics) using TB Green Premix Ex Taq II (Takara). Data were analysed using the 2^−△△Ct^ method, with GAPDH as the internal standard.

All qRT‐PCR primer sequences used in this study are listed in Table 1.

**TABLE 1:** The primers utilized in this study.

Gene	Primers
iNOS	Forward: CCTGCTTTGTGCGAAGTGTC
	Reverse: CCCAAACACCAAGCTCATGC
Arg‐1	Forward: TGTCCCTAATGACAGCTCCTT
	Reverse: GCATCCACCCAAATGACACAT
IL‐1β	Forward: CAGGATGAGGACATGAGCACC
	Reverse: CTCTGCAGACTCAAACTCCAC
IL‐10	Forward: GCTCTTACTGACTGGCATGAG
	Reverse: CGCAGCTCTAGGAGCATGTG
TNF‐α	Forward: AGCCGATGGGTTGTACCTTG
	Reverse: ATAGCAAATCGGCTGACGGT
VEGF	Forward: CAGGGACAGTTGCTTCTGGA
	Reverse: CAGGGACAGTTGCTTCTGGA
GAPDH	Forward: TGTCTCCTGCGACTTCAACA
	Reverse: GGTGGTCCAGGGTTTCTTACT

### 2.4 HE Staining

For HE staining, sections were dewaxed in graded alcohol, then subjected to staining using a solution comprising haematoxylin for cell nuclei and eosin for cytoplasm. Subsequent dehydration, transparency and sealing with neutral glue were carried out. Finally, specimens were visualized using a BX51 fluorescence microscope.

### 2.5 Immunohistochemistry

To assess macrophage polarization in the dental pulp during orthodontic force application, we examined the expression of macrophage polarization markers, iNOS and Arg‐1, in the pulp of the first left maxillary molar. Sections for IHC were deparaffinized and subjected to antigen retrieval using 0.25% trypsin (Solarbio) for 30 min at 37°C. This was followed by a 15‐min treatment with 3% hydrogen peroxide and a 1‐h blocking step with 5% normal goat serum (Solarbio) at room temperature. Primary antibodies were then applied to the sections overnight at 4°C. Subsequently, secondary antibodies were added, and the sections were counterstained with haematoxylin (Solarbio). Quantification was performed using ImageJ software.

All antibodies used for immunohistochemical staining in this study are listed in Table 2.

**TABLE 2:** The antibodies utilized in this study.

Antibodies	Source
iNOS	Proteintech, iNOS Polyclonal antibody, 18,985‐1‐AP
Arg‐1	Proteintech, Arginase‐1 Polyclonal antibody, 16,001‐1‐AP
F4/80	Proteintech, F4/80 Polyclonal antibody, 9414‐1‐AP
β‐Actin	Proteintech, beta Actin polyclonal antibody, 20,536‐1‐AP

### 2.6 Western blot

For Western blot analysis, RAW264.7 cells were lysed in 1× ice‐cold RIPA lysis buffer (NCM Biotech, WB3100) with EDTA‐Free Protease Inhibitor Cocktail (NCM Biotech, P001) and Phosphatase Inhibitor Cocktail 2 (Sigma‐Aldrich, P5726). After centrifugation, protein concentration was quantified using a BCA assay kit (Beyotime, P0012S) according to the manufacturer's instructions. Equal amounts of samples were separated by sodium dodecyl sulphate‐polyacrylamide gel electrophoresis and transferred to polyvinylidene difluoride membranes (IPVH00010, Millipore). Following a 2‐h blocking step with 5% non‐fat milk at room temperature, membranes were incubated with primary antibodies at 4°C overnight. After washing with TBS‐T, membranes were incubated for 1 h with horseradish peroxidase (HRP)‐conjugated secondary antibodies at room temperature, followed by further washes with TBS‐T. Protein bands were visualized using an Amersham Imager 600 (General Electric) with ECL Western Blotting Substrate (Biosharp, China). Densitometric quantification of protein bands was performed using ImageJ (National Institutes of Health).

All antibodies used for Western Blot in this study are listed in Table 2.

### 2.7 Immunofluorescence

MDPCs fixed with 4% paraformaldehyde were permeabilized with 0.1% Triton X‐100 in PBS for 20 min, rinsed with TBS and blocked with PBS containing 5% bovine serum albumin (BSA, Sigma‐Aldrich) for 1 h. The cells were then incubated with primary antibodies at 4°C overnight, followed by washing with PBS. Subsequently, the cells were stained with fluorophore‐conjugated secondary antibodies for 1 h at room temperature and 4′, 6‐diamidino‐2‐phenylindole (DAPI, Solarbio) for 5 min. The results were visualized using an IX73 inverted microscope (Olympus). Fluorescence intensity was quantified using ImageJ.

All antibodies used for immunofluorescence characterizations in this study are listed in Table 2.

### 2.8 Flow cytometric analysis

The cell surface markers of macrophages in mouse dental pulp were analysed by flow cytometry (BD Biosciences) according to the manufacturer's instructions. Briefly, after washing with PBS containing 3% FBS, the cells were incubated with monoclonal antibodies against mouse F4/80 (Biolegend, Cat. No. 123107) in the dark at 4°C for 1h; cells without pretreatment by any antibody were used as blank control. Subsequently, the cells were washed with PBS and analysed by flow cytometry.

### 2.9 ELISA

ELISA was conducted to quantitatively estimate cytokines, including IL‐1b, IL‐10, VEGF and TNF‐α, in the culture supernatants of MDPCs. Commercially available kits (from Elabscience) were employed as per the manufacturer's instructions. Supernatants were collected and stored at −80°C until analysis.

### 2.10 Statistical analysis

Data with a normal distribution were presented as mean ± standard deviation for each group, consisting of three or more independent samples. GraphPad Prism 9.0 software (GraphPad Software Inc.) was used for statistical analysis. Differences between two experimental groups were assessed using the Student's *t*‐test, while differences between three groups were analysed using one‐way analysis of variance (ANOVA). *p* < 0.05 was considered statistically significant.

## 3 RESULTS

### 3.1 Inflammation and hypoxia occur in pulp tissue during orthodontic tooth movement

**FIGURE 1:**
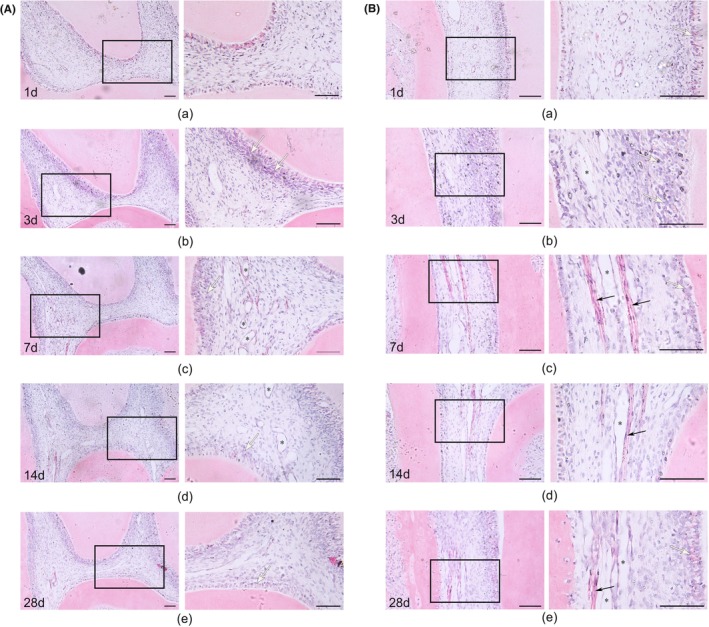
Inflammation and hypoxia occur in pulp tissue during orthodontic tooth movement. (A) HE staining of sections of the pulp cavity region in rats of 1, 3, 7, 14 and 28 days with orthodontic force. (B) HE staining of sections of the root canal region in rats of 1, 3, 7, 14 and 28 days with orthodontic force. White arrows indicate the arrangement of odontoblasts; Black arrows indicate the plasma proteins; * indicate the vascular dilatation. Scale bar = 100 μm.

During the application of orthodontic force, the pulpal tissue underwent significant changes over time. In the pulp cavity region, we observed a gradual disorganization of the odontoblasts arrangement (indicated by white arrows) from the 1 day group (Figure 1A[a]) to the 14 days group (Figure 1A[d]). At the same time, the pulpal characteristics changed, with an increase in inflammatory cell infiltration and showed significant pulp vacuolation and vascular dilatation (indicated by *). However, in the 28 days group, the disorganization of dentin cell arrangement and pulpal vacuoles subsided (Figure 1A[e]). Changes were more pronounced in the root canal region. We observed disorganized odontoblasts arrangement in the 3 days group (Figure 1B[b], indicated by white arrows). The root canals showed more pronounced vascularization, beginning with the 7 days group (Figure 1B[c]). A substance with light purple staining, suggesting the presence of plasma proteins (indicated by black arrows), indicated hypoxia and the onset of vascularization. This was accompanied by marked vasodilatation (indicated by *). The above indicates an inflammatory response of the pulp to mechanical force and vascularization is a compensatory change in the pulp in response to hypoxia. This is consistent with our previous study, which found elevated HIF‐1α and VEGF expression in the dental pulp.^12^

### 3.2 Macrophages polarize to the M2 type in pulp during orthodontic tooth movement

**FIGURE 2:**
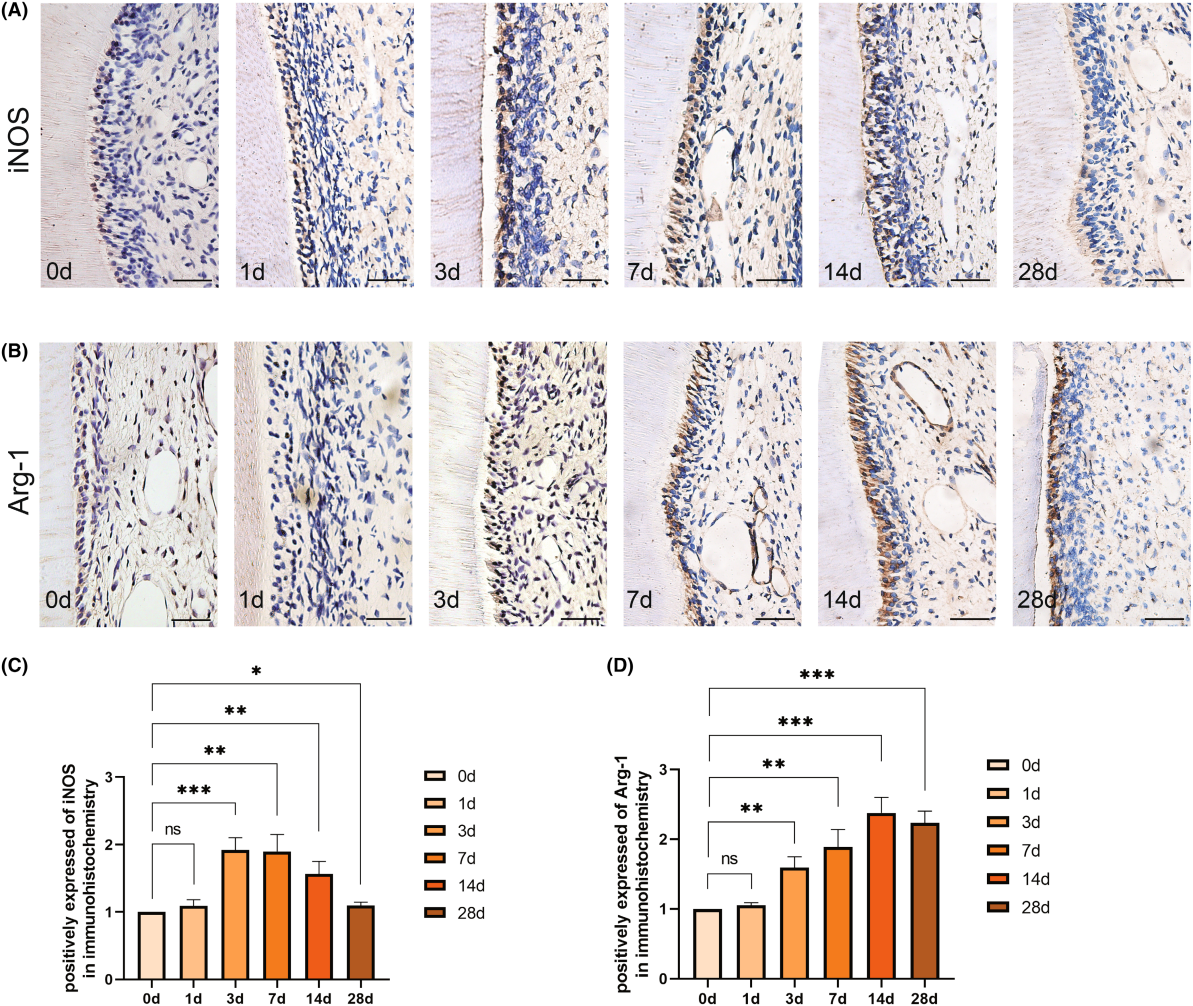
Macrophages polarize to the M2 type in pulp during orthodontic tooth movement. (A) Immunohistochemistry of regional sections of the pulp cavity regions of the upper left first molar in rats of 0, 1, 3, 7, 14 and 28 days with mechanical force. (B) Immunohistochemistry of sections of the root canal regions of the upper left first molar in rats of 0, 1, 3, 7, 14 and 28 days with mechanical force. (C) Positive expression of iNOS in immunohistochemistry of 0, 1, 3, 7, 14 and 28 days in pulp during orthodontic tooth movement. (D) Positively expressed of Arg‐1 in immunohistochemistry from 0, 1, 3, 7, 14 and 28 days in pulp during orthodontic tooth movement. Scale bar = 50 μm. *p < 0.05, **p < 0.01, ***p < 0.001, ns = no significance.

To clarify the polarization of the macrophages under mechanical force, we analysed the expression of iNOS, a marker of M1 polarization, and Arg‐1, a marker of M2 polarization in the pulp. Immunohistochemical results indicated that there was no significant difference in the expression of iNOS and Arg‐1 on the first day when compared to the control group. However, iNOS expression significantly increased at 3 days, followed by a declining trend after 7 days (Figure 2A,C). On the other hand, Arg‐1 expression displayed an upward trend after 3 days of force application and did not exhibit a decrease until the 28th day (Figure 2B,D). Furthermore, we observed that as the appearance of vascularization and inflammation resolution, iNOS expression decreased, and Arg‐1 expression correspondingly increased. This suggests a gradual polarization of macrophages towards M2 type, which may be involved in pulpal inflammation resolution and pulpal vascularization.

### 3.3 Hypoxia promotes the polarization of macrophages to the M2 type

**FIGURE 3:**
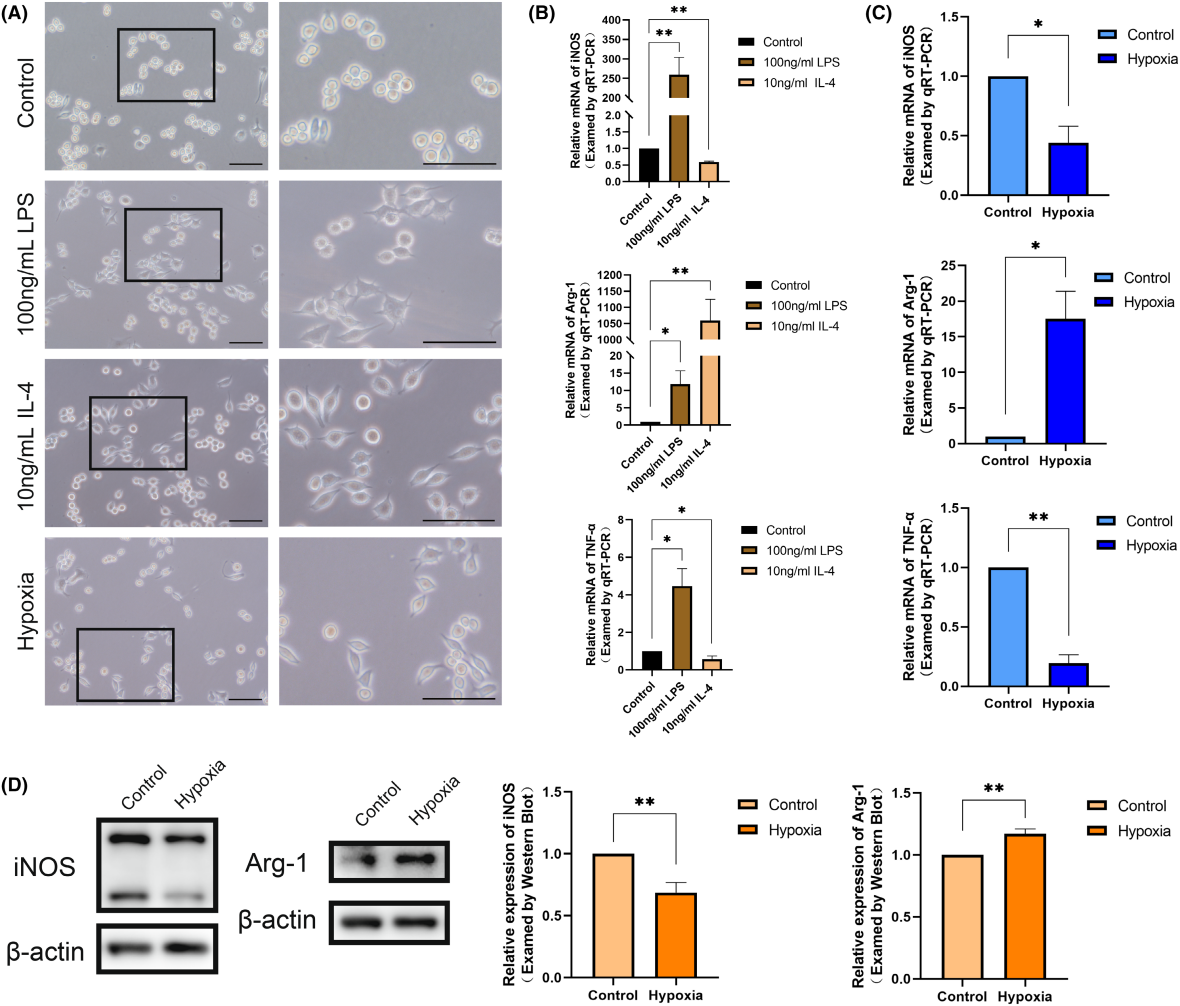
Hypoxia promotes the polarization of macrophages to the M2 type. (A) Morphology of normal cultured RAW264.7 cells and cultured with 100 ng/mL LPS and 10 ng/mL IL‐4 for 24 h. (B) The expression of iNOS, Arg‐1 and TNF‐α of normal cultured RAW264.7 cells and those cultured with 100 ng/mL LPS and 10 ng/mL IL‐4 for 24 h. (C) The mRNA level of iNOS, Arg‐1 and TNF‐αof RAW264.7 cells between normal culture and hypoxic culture. (D) The protein expression of iNOS and Arg‐1 of RAW264.7 cells between normal culture and hypoxic culture. Scale bar = 100 μm. *p < 0.05, **p < 0.01.

To further verify the effect of hypoxia on macrophage polarization, we, respectively, induced macrophage polarization towards pro‐inflammatory (M1) using 100 ng/mL LPS (24 h) and anti‐inflammatory (M2) using 10 ng/mL IL‐4 (24 h). We observed that LPS culture significantly increased the expression levels of iNOS and TNF‐α, indicating the classical activation of pro‐inflammatory macrophages (M1). Conversely, IL‐4 culture significantly increased the expression of Arg‐1 and decreased the expression of iNOS, signifying the selective activation of anti‐inflammatory macrophages (M2) (Figure 3B).

The normal cultured RAW264.7 cells exhibited round, rod‐shaped or irregular shapes with inconspicuous pseudopods. M1 direction‐induced RAW264.7 cells displayed a distinct ‘omelette’ morphology with more pseudopods and fork‐like changes. In contrast, M2 direction‐induced RAW264.7 cells adopted a stretched ‘spindle‐shaped’ appearance with fewer pseudopods (Figure 3A). The point is, RAW264.7 cells cultured with hypoxia for 24 h exhibited morphological similarities to anti‐inflammatory cells, appearing as pike‐shaped cells with fewer protruding pseudopods. In addition, the results of qRT‐PCR indicated that iNOS and inflammatory factor TNF‐α expression was decreased and Arg‐1 was increased in hypoxic cultured RAW264.7 cells (Figure 3C). Western Bolt also reflected the characteristics of anti‐inflammatory macrophages (M2) with decreased iNOS and increased Arg‐1 (Figure 3D). This is consistent with the macrophage polarization results we have observed in vivo. The above results further validate that mechanical force may promote macrophage M2 polarization through hypoxia in the pulp.

### 3.4 Inhibition of macrophages M2 polarization is detrimental to pulpal vascularization and inflammation resolution

**FIGURE 4:**
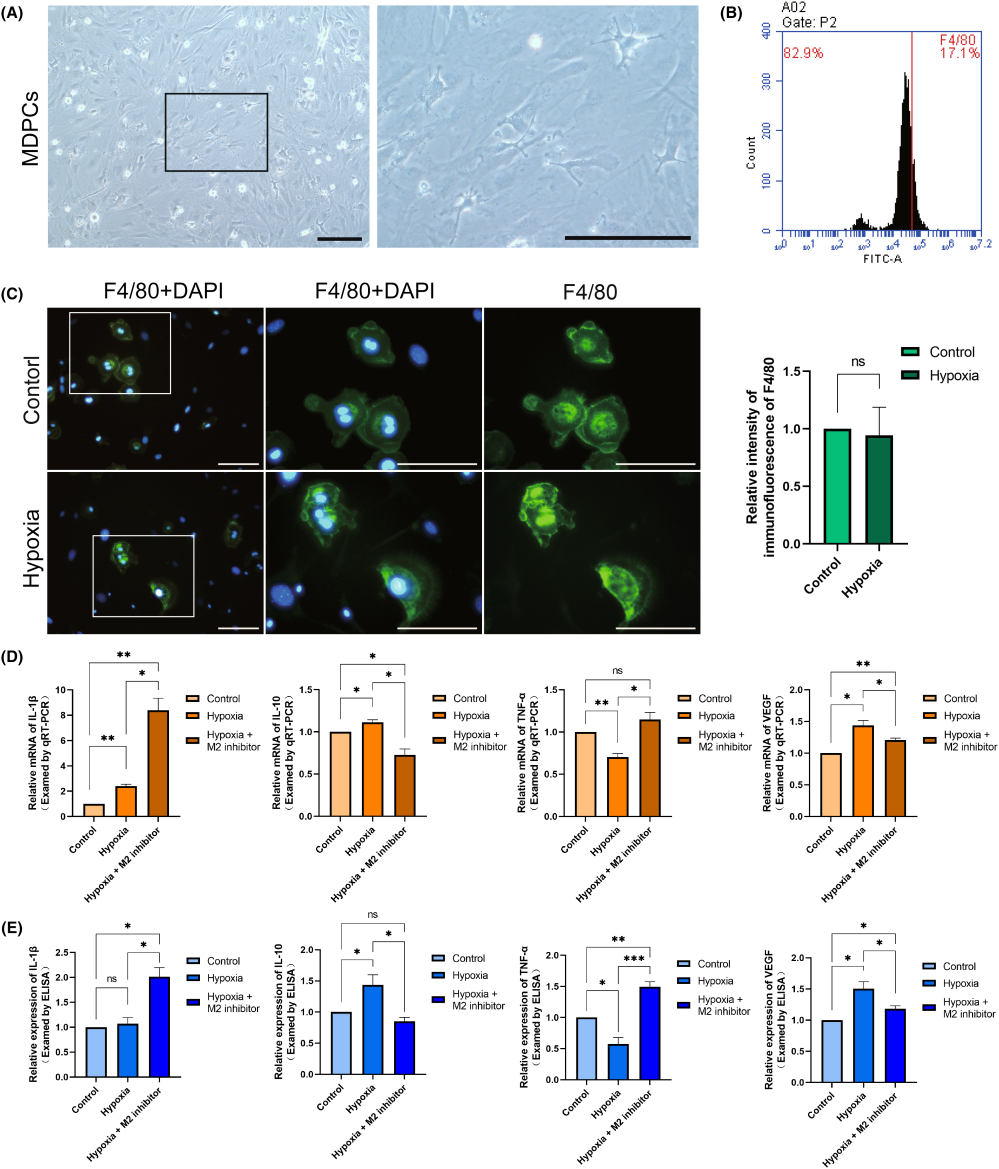
Inhibition of macrophages M2 polarization is detrimental to pulpal vascularization and inflammation resolution. (A) Cell morphology of mouse dental pulp cells. (B) Number of F4/80 positive cells in mouse dental pulp cells by flow cytometry. (C) The immunofluorescence staining and the level of F4/80 expression of control and hypoxic cultured groups. (D) The relative gene expression of IL‐1β, IL‐10, TNF‐α and VEGF in control, hypoxia, hypoxia + M2 inhibitor groups of MDPCs by qRT‐PCR. (E) The relative protein expression of IL‐1β, IL‐10, TNF‐α and VEGF in control, hypoxia, hypoxia + M2 inhibitor groups of MDPCs by ELISA. Scale bar = 100 μm. *p < 0.05, **p < 0.01, ***p < 0.001, ns = no significance.

We isolated MDPCs by enzyme digestion and microscopic techniques. Microscopically, MDPCs primarily comprised fibroblasts with elongated spindle‐like or stellate short protrusions, alongside a variety of pulp defence cells including macrophages, dendritic cells and lymphocytes (Figure 4A). Flow cytometric analysis of the cell surface marker F4/80 indicated that macrophages constituted approximately 15%–20% of the total cell quantity (Figure 4B). Subsequently, we subjected the extracted MDPCs to 24 h of hypoxic culture. The analysis of F4/80 immunofluorescence results suggested that the macrophage percentage remained relatively stable (Figure 4C). To examine the role of macrophages in pulp during orthodontic tooth movement, we cultured MDPCs with hypoxia after adding a selectively activated (M2) polarization inhibitor. By analysing the mRNA expression of IL‐1β, IL‐10, TNF‐α and VEGF, it revealed that inflammatory factors such as IL‐1β and TNF‐α exhibited an increase and the secretion of anti‐inflammatory factor IL‐10 and the VEGF decreased (Figure 4D). ELISA was also performed for the expression of the above factors in the culture supernatants. The results also showed an anti‐inflammatory trend (Figure 4E). These suggest that, for the entire pulp tissue, inhibition of macrophage M2 polarization would be detrimental to inflammatory regression and vascularization. Hypoxia induces macrophages to participate in pulpal vascularization and promoting inflammatory regression during orthodontic tooth movement.

## 4 DISCUSSION

Our examination of dental pulp tissues revealed distinct temporal changes in response to mechanical force, characterized by the presence of inflammatory cells, oedema and vascularization. This response is consistent with studies by Piero Römer,^20^ and Ikawa et al.^21^ reporting that hypoxia is a byproduct of orthodontic tooth movement, resulting from the compression of blood vessels in the periapical membrane. In addition, the gradual restoration of the inflammatory state is accompanied by increased vascularization and relief of hypoxia in pulp tissue.^22^ This suggests a role for hypoxia in initiating and maintaining the inflammatory cascade response. At the same time, the special structure of the teeth prevents the direct transmission of mechanical force to the pulpal tissues. Therefore, we propose that hypoxia may be a major stimulus in the pulp during orthodontic tooth movement.

We further investigated the response of macrophage polarization to mechanical force in vivo. Expression of the M1 marker iNOS was increased in the pulp during the early stage of orthodontic tooth movement. This confirms previous studies on the pro‐inflammatory role of classically activated macrophages (M1 macrophages).^23^ In contrast, the level of the M2 marker Arg‐1 gradually increased with the duration of force application. At the same time, iNOS expression decreased. This suggests a shift towards an anti‐inflammatory state of macrophages (M2) in the dental pulp.^24^ Comparing the process of polarization alteration with the progression of pulpal inflammation, we observed striking similarities between them. In the early stage of mechanical force application, M1 polarization was dominant, and a series of inflammatory reactions appeared in the pulp. As macrophages progressively exhibit M2 polarization, vascularization and resolution of the inflammatory responses in the pulp begin to occur. This reminds us that macrophage polarization may be involved in the regression of pulpal inflammation during orthodontic tooth movement.

Subsequently, we planned to elucidate the role of the macrophage in the dental pulp by blocking its polarization. Macrophages, as key effector cells, exhibit diverse metabolic reprogramming.^25^ They can play a role in promoting inflammation and orchestrating immune responses by M1‐type macrophages^26^ or promoting inflammation resolution and vascularization by M2‐type macrophages.^27^ It has been shown that M2 macrophage polarization can be driven by MEK/ERK,^28^ promoting the PPARγ/RA signalling axis, with pharmacological inhibitors like trametinib and panobinostat effectively blocking M2 polarization.^29^, ^30^ Of the two, trametinib can inhibit M2 polarization without affecting M1 polarization.^31^ Lastly, the addition of M2 polarization inhibitors to examine the impact of macrophage polarization on the overall dental pulp environment reveals the significant anti‐inflammatory and pro‐angiogenic effects of macrophages. Through the above experiments, we further emphasized the important role of macrophages in pulpal inflammation resolution and vascularization during orthodontic tooth movement.

However, it has been proposed that in vivo hypoxia promotes HIF‐1α accumulation, and elevated HIF‐1α expression promotes macrophage M1 polarization through activation of the HIF‐1α/pyruvate kinase M2 (PKM2) axis.^32^ This suggests that there may be more complex regulatory pathways for macrophage response to hypoxic stimuli in our body. More importantly, hypoxia is not a binary phenomenon. When investigating the effect of hypoxia on macrophage polarization, the effect of oxygen gradient on the results should be considered. In addition, in order to clearly further define the role of macrophage M2 polarization in vivo, it may be necessary to further establish knockout animal models of macrophage M2 polarization‐related genes. And further studies may be needed to determine the underlying mechanisms of how polarized macrophages regulate dental pulp homeostasis.

**FIGURE 5:**
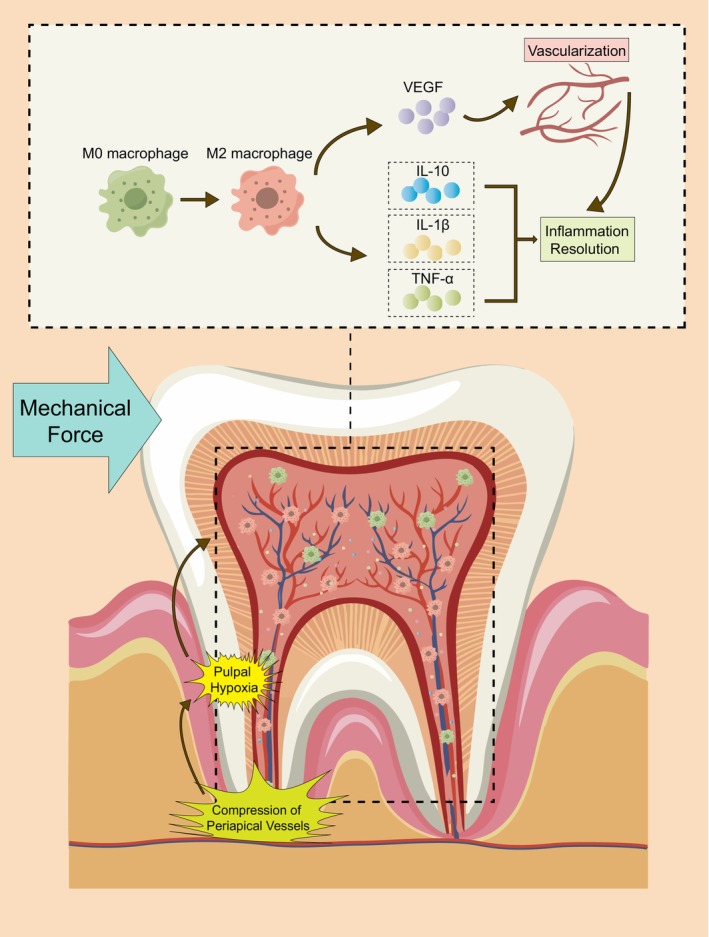
Schematic Overview of macrophage M2 polarization promotes pulpal inflammation resolution during orthodontic tooth movement. Under mechanical force, the periodontal tissues, especially the periapical vessels of the tooth, are compressed. The shortage of blood supply in the pulp leads to hypoxia. Hypoxia further promotes inflammatory responses. At this point, macrophages in the dental pulp are activated by hypoxia and induced to become M2‐type macrophages. This reduces the expression levels of IL‐1β and TNF‐α and increases the expression of IL‐10 in the pulp tissue. It also promotes the secretion of VEGF from the pulp tissue, which further promotes inflammation resolution through vascularization.

In conclusion, our study illustrates the complex interplay between mechanical force, hypoxia, macrophage polarization, and their profound influence on dental pulp tissue. We found that pulpal macrophages gradually shift towards an anti‐inflammatory state (M2) in orthodontic teeth. Hypoxia can induce macrophages to promote the expression of anti‐inflammatory factors and VEGF by dental pulp cells. This transformation in polarization status significantly contributes to the regression of pulpal hypoxia and inflammation (Figure 5). These findings provide a further cellular foundation for maintaining orthodontic dental health and new directions for investigation of the role played by macrophage polarization between hypoxia and inflammation.

## AUTHOR CONTRIBUTIONS

**Jichang Li:** Conceptualization (equal); data curation (lead); formal analysis (lead); investigation (lead); methodology (lead); project administration (lead); supervision (equal); visualization (lead); writing – original draft (lead); writing – review and editing (lead). **Huiying Ren:** Data curation (supporting); investigation (supporting). **Zijie Zhang:** Data curation (supporting); formal analysis (supporting); writing – review and editing (supporting). **Jin Zhang:** Conceptualization (supporting); project administration (supporting). **Fulan Wei:** Funding acquisition (lead); project administration (supporting); supervision (lead); writing – review and editing (supporting).

## FUNDING INFORMATION

This research was funded by the National Natural Science Foundation of China (82071080), the Fundamental Research Funds for the Central Universities (2022JC017) and the Academician Laboratory of Immune and Oral Development & Regeneration, Dalian Medical University (2021002).

## CONFLICT OF INTEREST STATEMENT

The authors declare that they have no competing interests.

## CONSENT FOR PUBLICATION

Not applicable.

## Data Availability

All data presented in the current study are available from the corresponding author on reasonable request.
